# Prenatal metal mixtures and sex-specific infant negative affectivity

**DOI:** 10.1097/EE9.0000000000000147

**Published:** 2021-04-02

**Authors:** Whitney Cowell, Elena Colicino, Yuri Levin-Schwartz, Michelle Bosquet Enlow, Chitra Amarasiriwardena, Syam S. Andra, Chris Gennings, Robert O. Wright, Rosalind J. Wright

**Affiliations:** a Department of Environmental Medicine and Public Health, Icahn School of Medicine at Mount Sinai, New York, NY; b Department of Psychiatry, Boston Children’s Hospital, Boston, MA; c Department of Psychiatry, Harvard Medical School, Boston, MA; d Institute for Exposomic Research, Icahn School of Medicine at Mount Sinai, New York, NY; e Kravis Children’s Hospital, Department of Pediatrics, Icahn School of Medicine at Mount Sinai, New York, NY.

## Abstract

Prenatal exposure to metals has been associated with a range of adverse neurocognitive outcomes; however, associations with early behavioral development are less well understood. We examined joint exposure to multiple co-occurring metals in relation to infant negative affect, a stable temperamental trait linked to psychopathology among children and adults.

**Methods:**

Analyses included 308 mother-infant pairs enrolled in the PRISM pregnancy cohort. We measured As, Ba, Cd, Cs, Cr, Pb, and Sb in urine, collected on average during late pregnancy, by ICP-MS. At age 6 months, we assessed negative affect using the Infant Behavior Questionnaire—Revised. We used Weighted Quantile Sum (WQS) regression with repeated holdout validation to estimate the joint association between the metals and global negative affectivity, as well as four subdomains (Fear, Sadness, Distress to Limitations, and Falling Reactivity). We also tested for a sex interaction with estimated stratified weights.

**Results:**

In adjusted models, urinary metals were associated with higher scores on the Fear scale (β_WQS_ = 0.20, 95% confidence interval [CI]: 0.09, 0.30), which captures behavioral inhibition, characterized by startle or distress to sudden changes in the environment and inhibited approach to novelty. We observed a significant sex interaction (95% CI for the cross-product term: −0.19, −0.01), and stratified weights showed girls (61.6%) contributed substantially more to the mixture effect compared with boys (38.4%). Overall, Ba contributed the greatest mixture weight (22.5%), followed by Cs (14.9%) and As (14.6%).

**Conclusions:**

Prenatal exposure to metals was associated with increased infant scores on the temperamental domain of fear, with girls showing particular sensitivity.

Key words: Prenatal; Metals; Mixtures; Temperament; Infancy; Negative affect

## Abstract 2

Supplemental Digital Content is available in the text.

**What this study adds**

We investigated maternal late-pregnancy urinary metals in relation to infant negative affectivity, a core temperamental trait, among participants enrolled in the PRISM pregnancy cohort. We show, for the first time, the application of two recently developed weighted quantile sum (WQS) regression extensions for (1) improving the stability of estimates across training and test partitions, and (2) modeling interactions and estimating stratified weights. We found increasing exposure to metals was associated with increased fear and behavioral inhibition, with notable sex differences.

## Introduction

Temperament encompasses biologically based interindividual differences in reactivity and self-regulation to internal, social, and environmental cues that are longitudinally stable and consistent across situations.^1^ Temperamental traits are observable in infancy, with early maladaptive displays serving as risk factors for behavioral and physical problems during later childhood and into adult life.^2^ Temperament is theorized to have both genetic and environmental origins and may be influenced by exposures during the prenatal period, when sympathetic and parasympathetic processes, which provide a physiological basis for temperamental features, begin to develop.^3^ Autonomic differentiation and neurologic maturation are reflected in fetal motor activity, heart rate, and heart rate variability, which have been shown to predict regulatory functions during infancy and childhood. For example, stable individual differences in activity, a core temperamental trait that can be measured prenatally, are identifiable in the fetus^4^ and track with activity levels during the neonatal,^5^ infant,^6^ and toddler^7^ periods. Fetal motor activity has also been linked to specific behavioral outcomes at 6 months of age, with more active fetuses rated as fussier, unadaptable, and unpredictable in infancy.^8^ More recent research made possible by advances in monitoring technology has linked fetal heart rate and heart rate reactivity with irritability, fear, and inhibition during infancy and childhood.^9,10^

Limited research suggests that environmental exposures during pregnancy may influence the development of temperamental features. For example, experimental studies have shown that the fetus responds, as indicated by reduced movement and changes in heart rate, to maternal cigarette smoking during sessions.^11,12^ Longitudinal research also supports that prenatal exposure to toxic substances (cocaine, tobacco, alcohol, marijuana) is associated with temperamental features at age 4 months, including increased distress to novelty and limitations.^13^ However, we are not aware of research that has examined other environmental exposures in relation to infant temperamental traits.

Various lines of research support that prenatal exposure to metals is negatively related to neurodevelopment and may influence infant temperament. Most metals occur naturally in the Earth’s crust and are released into the environment with natural weathering and erosion. Metals migrate into the soil, groundwater, and air leading to the potential for human exposure through inhalation or ingestion of contaminated food, water, or dust. As detailed below, other sources of exposure to metals include cigarette smoking and contact with metal-containing consumer products. General population exposure to arsenic (As) occurs through consumption of contaminated water and food, with seafood considered the predominant dietary source, although As in seafood generally has low toxicity.^14^ Other contributors to dietary intake of As, including inorganic species, include rice, poultry, mushrooms, and juice.^14^ Exposure to trace levels of barium (Ba) has historically occurred through drinking water; however, the past few decades have seen an increase in industrial processes that release Ba into groundwater (petroleum drilling, hydraulic fracturing, unconventional shale gas wells), which has led to an increased propensity for human exposure.^15,16^ The primary source of exposure to cadmium (Cd) is cigarette smoking, as tobacco leaves accumulate a high level of Cd from soil.^17^ Among nonsmokers, diet contributes the greatest to Cd exposure, with leafy vegetables, grains, and seeds typically having elevated levels.^17^ The general population is exposed to low levels of antimony (Sb) in ambient air, drinking water, and food, with some consumer products such as PET water bottles, polyester textiles, and other products containing antimony flame retardants also considered sources of exposure.^18,19^ Biomonitoring studies have found Sb levels are higher among older adults, omnivores (versus vegetarians), and tobacco users.^20^ Exposure to lead (Pb) occurs from drinking contaminated water (e.g., from lead pipes), ingestion or inhalation of dust contaminated from lead paint, and ingestion through produce that has taken up lead from the soil.^21^ Pb can also enter food from glazed pottery, ceramic dishes, and leaded-crystal glassware.^21^ Finally, human exposure to cesium (Cs) and chromium (Cr) occurs through inhalation, drinking water, or consumption of contaminated food/supplements, although Cs levels in the air and water are generally very low; exposure to Cr can additionally occur via. cigarette smoking.^22–24^

Several metals have been shown to readily cross the placenta^25,26^ and fetal exposure, even at low levels, can interfere with the course of neurodevelopment processes, with consequences for cognitive, emotional, and behavioral outcomes during childhood.^27–29^ Pb, As, and Cd are well-established neurotoxicants,^29,30^ although less research has investigated their joint effects. In one experimental murine study, prenatal exposure to these metals was associated with disruption of the blood-brain barrier, dose-dependent deposition in the developing brain, and altered neuron morphology.^31^ Exposed offspring also exhibited behavioral disturbances and learning-memory deficits, with evidence of synergistic effects between metals.^32^ Metals may also induce developmental neurotoxicity through inflammation or oxidative stress-mediated pathways.^33^ For example, in rats, barium (Ba) exposure during pregnancy has been shown to induce oxidative stress in the brains of both dams and pups, resulting in histological damage.^34^ Likewise, Pb and Cd have been shown to not only induce oxidative stress^35,36^ but also significantly decrease the capacity of antioxidant enzymes.^37,38^ In turn, oxidative stress is associated with cell death, including in the brain^39^ and is implicated in the pathogenesis of adverse neurodevelopmental outcomes.^40,41^ In contrast to these metals, little research has investigated the developmental toxicity of Cr, Cs, or Sb.

With regard to behavior, prior research has reported associations between prenatal exposure to metals and increased negative emotionality during infancy,^42^ reduced regulatory capacity among toddlers,^43^ and more emotional problems during childhood.^44^ Negative affect—the tendency to experience sadness, fear, anger, irritability, and anxiety—may be particularly important for developmental outcomes, as it is a relatively stable temperamental trait across the life course, with high trait negative effect during infancy being a strong predictor of psychopathology in childhood.^45^ High negative effect is also an established precursor of the adult personality factor of neuroticism,^2^ which, notably, has been associated with childhood metals exposure.^46^

Sexually dimorphic brain circuitry and morphology is well established, and sex-specific properties of the blood-brain barrier, transporter functions, metabolism, and detoxification result in sex differences in metal uptake and toxicity.^47–49^ Further, many neurodevelopmental disorders present with a sex bias,^50^ and epidemiologic research has demonstrated significant metal by sex interactions across a range of neurocognitive outcomes. For example, prenatal exposure to Pb has been negatively associated with neuropsychological development at age 30 months^51^ and increased attention-concentration problems during adolescence among boys but not girls.^31^ Other research has found prenatal As exposure is associated with decreased verbal and full-scale IQ among girls, but not boys, at age 5 years.^52^ However, little research has considered sex differential associations between prenatal metals and temperamental or other neurobehavioral outcomes.

In the present study, we examined prenatal exposure to a mixture of seven metals measured in urine in relation to global and subdomains (fear, sadness, distress reactivity, and arousal recovery) of infant negative effect assessed at 6 months of age in the PRogramming of Intergenerational Stress Mechanisms (PRISM) pregnancy cohort. Considering the effect of these metals jointly is critical, as interactions may change the kinetics and dynamics of the mixture components, ultimately resulting in altered toxicity.^53^ Further, given accumulating evidence that sex may moderate associations between prenatal metals exposure and a range of neurodevelopmental and other health outcomes,^54–60^ we examined differences by infant sex.

## Methods

### Study Sample

PRISM is a prospective pregnancy cohort designed to examine the health effects of prenatal and early life psychosocial and environmental exposures. Beginning in 2011, pregnant women were recruited from prenatal clinics in Boston and New York City. Women were considered eligible if they were English or Spanish speaking, 18 years or older, and pregnant with a singleton. Exclusion criteria included maternal intake of ≥7 alcoholic drinks per week before pregnancy, any alcohol intake after pregnancy recognition, or HIV+ status. Recruitment remains ongoing; at the time of metals analysis, 926 enrolled, eligible mothers had delivered a live newborn with no significant congenital anomalies noted at birth that could impact further participation. Metals and creatinine were measured in a subset of urine samples collected during pregnancy (N = 348). Among these 348 participants, infant temperament was evaluated at age 6 months for 309 infants. We additionally excluded one mother-infant dyad missing key covariate information, resulting in a final analytic sample of N = 308. Children included in the analytic sample were enrolled between July 2011 and February 2018. Written informed consent was obtained from women before study participation in their preferred language. All study procedures were approved by the Institutional Review Boards at the Brigham and Women’s Hospital or the Icahn School of Medicine at Mount Sinai.

### Urinary metals and creatinine

During pregnancy (mean: 32 weeks, range: 15–40 weeks), women were instructed to collect a spot urine sample in their home on the morning of a scheduled study visit. Samples were kept cool in the participant’s freezer until transport to the PRISM laboratory on the day of collection. Immediately upon arrival, the urine samples were thawed, aliquoted, and stored at −20°C. Samples (200 µL) were diluted to 10 ml with a solution containing 0.05% Triton X-100, 0.5% nitric acid, and mixed internal standard before analysis of metal concentrations on an inductively coupled plasma-mass spectrometer-triple quadrupole (ICP-MS) instrument (Agilent 8800-QQQ). The seven metals included in the present analysis were selected from the set of metals that are measured by the Children’s Health Exposure Analysis Resource (CHEAR) urine metals panel (Al, As, Ba, Cd, Co, Cr, Cs, Cu, Mo, Mg, Mn, Ni, Pb, Sb, Se, Sn, Tl, V, Zn). Among this panel of metals, six (Al, Mg, Mo, Sn, Tl, V) were added to the panel partway through PRISM metals analysis and were thus measured in only 77% of samples. These six metals were excluded from analyses to maximize our sample size, which is constrained to those infants with available data on temperament at age six months. Of the remaining metals, we excluded essential nutrients (Co, Cu, Mn, Ni, Se, Zn) as our statistical approach, weighted quantile sum (WQS) regression, is inherently unidirectional, leaving seven metals (As, Ba, Cd, Cr, Cs, Pb, Sb) for inclusion in this analysis. Urine creatinine was measured using a well-established colorimetric method (limit of detection [LOD]: 0.3125 mg/dL).^61,62^ Quality control measures for metals and creatinine analysis have been previously described in detail.^63^ The LODs and the number of samples below the LOD are provided in Table 2. We replaced metal concentrations below the LOD with the LOD divided by the square root of two. Our approach to nondetects likely has minimal impact given that exposure levels are decided in the mixture analysis.

**Table 1.** PRISM sample characteristics overall and by infant sex

	Overall (n = 308)	Boys (n = 171)	Girls (n = 137)	*P *^a^
Maternal age (years)	28.9 ± 5.7	28.8 ± 5.6	29.0 ± 5.9	0.77
Race/ethnicity				0.73
White, non-Hispanic	39 (12.7)	24 (14.0)	15 (11.0)	
Black, Black-Hispanic	165 (53.6)	87 (50.9)	78 (56.9)	
Hispanic, non-Black	95 (30.8)	55 (32.2)	40 (29.2)	
Other	9 (2.9)	5 (2.9)	4 (2.9)	
Less than high school education	125 (40.5)	66 (38.6)	59 (43.1)	0.43
Smoke exposure^b^	121 (39.3)	67 (39.2)	54 (39.4)	0.96
Infant age at assessment (months)	6.5 ± 1.6	6.4 ± 1.5	6.5 ± 1.7	0.75
Gestational week of urine collection	31.1 ± 6.0	30.9 ± 6.1	31.2 ± 5.9	0.63
Maternal urinary creatinine (mg/dL)^c^	108.57 (90.45)	107.56 (90.45)	107.81 (90.45)	0.46
IBQ-R scales				
Global Negative Affectivity	3.14 ± 0.68	3.11 ± 0.64	3.18 ± 0.72	0.34
Fear	2.79 ± 1.03	2.62 ± 0.96	2.99 ± 1.08	0.002
Sadness	3.16 ± 0.88	3.17 ± 0.85	3.16 ± 0.92	0.95
Distress to Limitations	3.68 ± 0.91	3.66 ± 0.92	3.69 ± 0.91	0.78
Falling Reactivity	5.07 ± 1.03	5.02 ± 1.00	5.12 ± 1.06	0.40

Values are mean ± standard deviation or n (%) unless otherwise noted.

^a^*P* values are from *t*-tests (continuous variables) or chi-square tests of homogeneity (categorical variables) examining differences in covariates and IBQ-R scores by sex unless otherwise noted.

^*b*^Defined as maternal cigarette, cigar, or pipe smoking during pregnancy or exposure to environmental tobacco smoke for 1 hour or more per week during pregnancy.

^c^Summary statistics are geometric mean (interquartile range), and *P* values are from Wilcoxon Rank Sum tests.

IBQ-R indicates Infant Behavior Questionnaire-Revised.

**Table 2.** Concentration of maternal late-pregnancy urinary metal concentrations (ng/ml) in the PRISM cohort (n = 308)

	25th percentile	50th percentile	75th percentile	Mean ± SD	N < LOD (%)	LOD Boston	LOD New York
Antimony	0.08	0.12	0.17	0.15 ± 0.12	61 (19.8)	0.077	0.077
Arsenic	5.89	9.99	20.55	18.86 ± 27.01	1 (0.3)	0.259	0.323
Barium	1.66	2.80	4.76	3.86 ± 3.46	3 (1.0)	0.563	0.262
Cadmium	0.11	0.21	0.39	0.31 ± 0.33	42 (13.6)	0.028	0.075
Chromium	0.48	0.62	0.84	0.84 ± 2.21	33 (10.7)	0.288	0.823
Cesium	3.16	4.84	6.85	5.54 ± 3.47	0 (0.0)	0.103	0.065
Lead	0.49	0.70	0.98	0.80 ± 0.59	1 (0.3)	0.097	0.080

LOD indicates limit of detection.

### Infant negative affect

Mothers completed the Infant Behavior Questionnaire-Revised (IBQ-R) during an in-person visit when infants were approximately 6 months of age (6.4 ± 1.6).^2^ The IBQ-R is one of the most frequently used measures for assessing temperament in infants aged 3 to 12 months old,^64^ including among English- and Spanish-speaking families.^65,66^ Mothers reported the frequency of 191 specific infant behaviors and reactions to concrete situations across the previous 1 or 2 weeks. A trained research assistant read each item to the mother and recorded the mother’s response, which was rated on a 7-point Likert-type scale (1 = never, 7 = always), on the questionnaire form. We calculated 14 temperament scales following IBQ-R scoring criteria, including four scales (Fear, Distress to Limitations/Frustration, Sadness, Falling Reactivity/Rate of Recovery) that have been shown to load on an overarching dimension of Negative Affectivity in factor analytic studies.^2^ The Fear scale comprises 16 items and assesses inhibited approach to novel objects or other stimuli, as well as distress in social situations that involve novelty or uncertainty. The Distress to Limitations scale includes 16 items and assesses the propensity to distress (i.e., fussing, crying) during caretaking activities or when confined by place or position. The 14-item Sadness scale assesses lower mood and activity related to personal suffering, object loss, or inability to perform a desired action. Finally, the 13-item Falling Reactivity scale assesses the rate of infant recovery from peak arousal, distress, or excitement and reflects the infant’s own abilities to regulate state. We examined each of these scales, as well as a global Negative Affectivity composite score. Consistent with published scoring guidelines, the composite score was calculated by averaging the mean scores of each of the four subscales.^67^ Across scales, higher scores indicate greater negative affectivity, with the exception of Falling Reactivity, which is reverse scored when calculating the composite measure.

### Covariates

We *a priori* selected infant exact age (continuous, in days) at IBQ-R assessment as a covariate to account for variability related to typical changes in temperament that occur throughout the first year of life.^68,69^ Likewise, we adjusted for gestational week of urine collection (continuous in weeks) and urinary creatinine (continuous, mg/dL) to account for variability arising from pregnancy-related physiological changes and urine dilution that may influence metals concentrations. We used directed acyclic graph (DAG) theory to select other covariates for inclusion based on substantive knowledge, review of prior studies, and assumed conditional dependencies between variables (see eFigure 1; http://links.lww.com/EE/A131). In addition to the three precision variables selected *a priori*, final models were adjusted for maternal age (continuous, in years), race/ethnicity (non-Hispanic White or other [reference] vs. Black/Hispanic-Black vs. Hispanic/non-Black), smoke exposure during pregnancy (yes vs. no as described below), and level of education (less than high school degree vs. high school degree or more) as an indicator of socioeconomic status. Maternal race/ethnicity, age, and education were self-reported during an in-person interview during mid- to late pregnancy. Information on cigarette, cigar, or pipe smoking and the degree to which women were exposed to environmental tobacco smoke during pregnancy was ascertained during pregnancy and again during the postpartum period. We considered women to be smoke exposed if they reported either being an active smoker or were exposed to environmental tobacco smoke for 1 hour or more per week during pregnancy. In sensitivity analyses, we explored the associations of (1) metals exposure and (2) infant negative affectivity, with maternal fish intake, which was collected during the 2nd and 3rd trimesters for a subset of 148 participants (48%). Specifically, participants were asked the frequency with which they ate the following fish: fishsticks/friedfish/fish sandwich, tuna, salmon, halibut, trout, mackerel, herring, sardines, or other whitefish. We summed the frequency of consumption (in weeks) across the nine fish categories for the 2nd and 3rd trimester separately before taking the average across trimesters. We then created a categorical variable to reflect the average frequency of fish consumption across the 2nd and 3rd trimesters as follows: less than once per week (n = 80, 54%), 1–2 times per week (n = 34, 23%), or more than 2 times per week (n = 34, 23%). We created these categories based on the distribution of the data and to allow a reasonable sample size in each category.

### Statistical analyses

We visually examined the distribution of each metal, covariate, and negative affect scale, calculated descriptive statistics for the sample overall and stratified by infant sex, and examined Spearman rank correlations between the metals to understand potential collinearity. We used chi-square tests for homogeneity and t-tests to assess potential differences in sample characteristics between (1) boys versus girls and (2) participants included in the analytic sample versus those enrolled but excluded due to incomplete exposure or outcome data.

We used weighted quantile sum (WQS) regression to estimate the joint association of the mixture of all seven metals with scores on the global Negative Affectivity scale and each subscale (Fear, Sadness, Distress to Limitations, Falling Reactivity) in separate models. WQS regression is a multi-step approach for minimizing multicollinearity and evaluating the association between a set of correlated coexposures and an outcome.^70,71^ In the first step, empirical weights are simultaneously estimated across bootstrapped samples of the initial data (40% training) based on the association of each component with the outcome. The mean weight is then applied to exposure quantiles, here deciles, and summed to create a unidirectional weighted index, which is regressed on the outcome to estimate the overall mixture effect using the remaining data (60% validation). The coefficient is interpreted as the combined effect of the mixture, whereas the weight assigned to each metal reflects its relative contribution to the overall effect. To address generalizability of the estimates across training and testing sets, we implemented repeated holdout validation to randomly partition the data 100 times and repeat WQS regression on each set to produce a distribution of validated results.^72^ We report the mean across simulated distributions as the final estimate for metal weights and express effect estimates as the difference in Negative Affectivity domain scores per decile increase in the WQS metal mixture index. We hypothesized that increased metals exposure would be associated with higher scores on the global Negative Affectivity scale, and the Fear, Sadness, and Distress to Limitations subscales; therefore, we constrained parameter estimates to be positive for these models. Conversely, we hypothesized the association with Falling Reactivity, which reflects how quickly an infant recovers from a state of high arousal, would be inverse, and thus constrained the parameter estimates for this model to be negative. We confirmed these assumptions in separate regression models examining each metal individually (see eTable 3; http://links.lww.com/EE/A131). Given prior research showing sex-differential associations between metals exposure and a range of neurodevelopmental and behavioral outcomes, we examined whether the contribution of mixture components varied by infant sex by estimating sex-stratified weights. We also tested for different slopes between sexes by including a cross-product term between the mixture index and infant sex in each model. Finally, due to potential concerns of confounding by fish intake, we examined the frequency of maternal fish consumption during pregnancy (<1× per week, 1–2× per week, >2× per week) in relation to (1) metals exposure (continuous in ng/mL), and (2) infant negative affectivity (continuous), using separate Kruskal-Wallis tests for each metal and for each negative affect domain.

## Results

### Study sample

Women were on average 28 years old at delivery, and the majority self-identified as Black/Black-Hispanic (54%) or Hispanic/non-Black (31%), with the remainder of the sample identifying as White/non-Hispanic (13%) or other race/ethnicity (3%). Approximately 40% of women had less than a high school education, and 39% reported smoking and exposure to smoke (cigarette, cigar, or pipe) for 1 hour or more per week during pregnancy. Table 1 provides additional details of the study sample. We observed no differences in sociodemographic characteristics or maternal urinary metal concentrations by infant sex. The IBQ-R Negative Affectivity composite and subdomain scores were approximately normally distributed (Table 1). Consistent with prior studies, girls (2.99 ± 1.08) scored higher on the Fear scale compared with boys (2.62 ± 0.96, *P* = 0.002)^73^; no other significant sex-differences in the composite or subdomain scores were observed. The included sample was comprised of more Black/Black-Hispanic (54% vs. 40%), fewer White/non-Hispanic (13% vs. 18%), and fewer Hispanic/non-Black (31% vs. 37%) participants compared with the excluded sample (*P* = 0.001) and comprised more women who reported smoke exposure during pregnancy (39% vs. 25%, *P* < 0.001). The difference in smoke exposure may reflect differences in race/ethnicity, as Black/Black-Hispanic women were more likely to report smoke exposure (38%) compared with Hispanic/non-Black women (28%, *P* < 0.0001). We observed no other differences in sociodemographic or lifestyle characteristics, metals exposure, or temperament scores between the included versus excluded sample (see eTable 1; http://links.lww.com/EE/A131).

### Metals exposure

On average, maternal urine was collected at 32 weeks gestation (minimum: 14, maximum: 40). Urinary concentrations of As, Ba, Cd, Cr, Cs, Pb, and Sb followed log-normal distributions; summary statistics are provided for each metal in Table 2, and box plots presenting distributions are provided in the eFigure 2; http://links.lww.com/EE/A131. Urinary metal concentrations were positively correlated with each other, with Spearman coefficients ranging from 0.16 to 0.64 (*P* ≤ 0.001–0.008) (see eTable 2; http://links.lww.com/EE/A131). Based on 2015–2016, urinary metals data reported by the National Health and Nutrition Examination Study (NHANES), women enrolled in PRISM had higher concentrations of every metal compared with the general US population overall and to the US female population (see eFigure 3; http://links.lww.com/EE/A131), with the exception of Cr, which is not measured by NHANES.^74^ With regard to fish intake, there was a trend toward increasing total As levels with increasing fish consumption (*P* = 0.107). We did not detect associations between fish intake and the other metals considered (all *P* > 0.2), which are not typically linked to fish consumption.

### Metal mixture and infant negative affectivity

**Figure 1.**
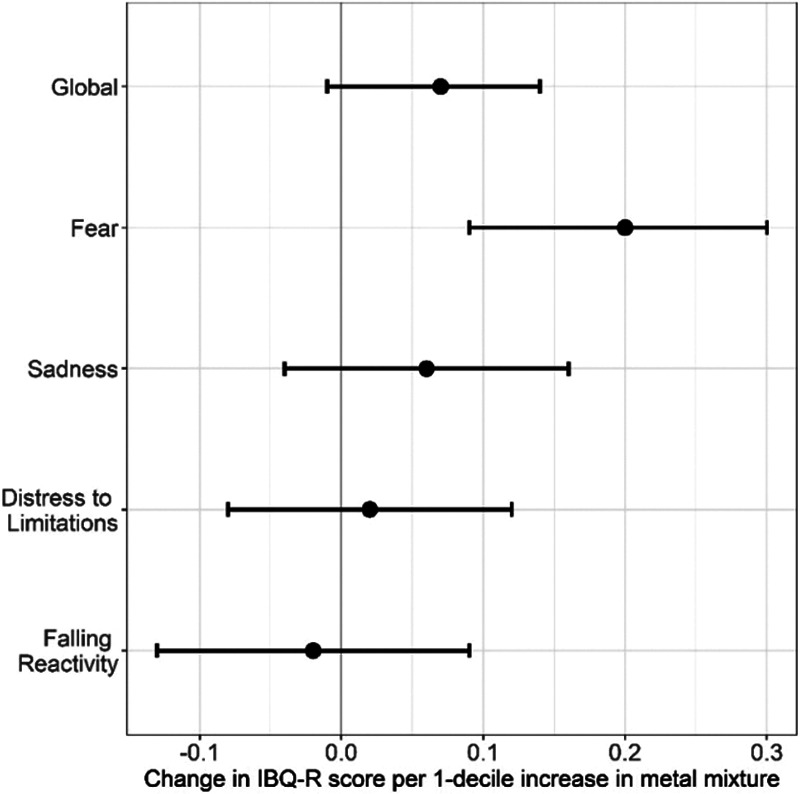
Change in IBQ-R negative affectivity global and subdomain scores for a 1-decile increase in the maternal metals Weighted Quantile Sum Index. Plotted points are beta coefficients, and lines are 95% confidence intervals. Models are adjusted for: infant age at IBQ-R assessment (days), gestational week of urine collection (weeks), creatinine (mg/dL), maternal age (years), race/ethnicity (non-Hispanic White or other vs. Black/Hispanic-Black vs. Hispanic/non-Black), smoke exposure (yes vs. no as described in manuscript), and maternal education (less than high school degree vs. high school degree or more). IBQ-R indicates Infant Behavior Questionnaire-Revised.

**Figure 2.**
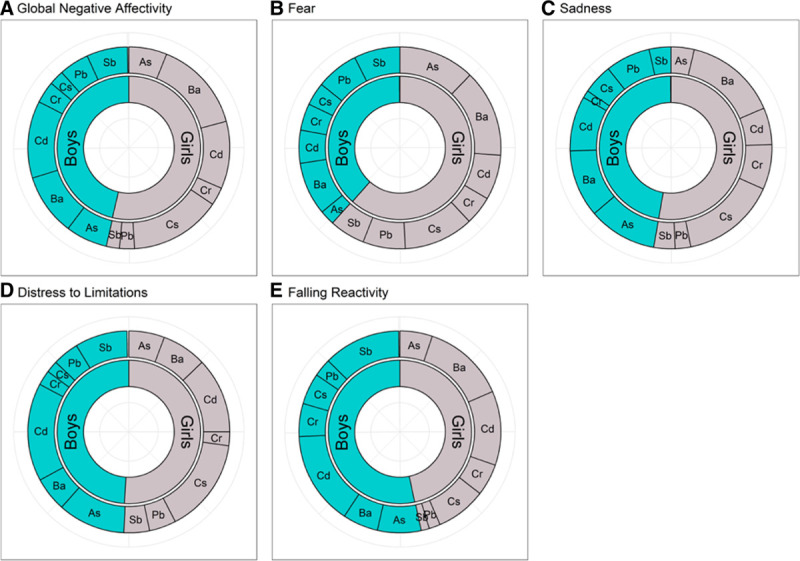
Metal mixture weights for the global and subdomains of infant negative affect (Fear, Sadness, Distress to Limitations, Falling Reactivity), stratified by infant sex. Values represent the mean weights across 100 repeated holdouts. Higher negative affect is characterized by higher scores on the global negative affectivity domain score and on the Fear, Sadness, and Distress to Limitations scales, and lower scores on the Falling Reactivity scale. Models are adjusted for: infant age at IBQ-R assessment (days), gestational week of urine collection (weeks), creatinine (mg/dL), maternal age (years), race/ethnicity (non-Hispanic White or other vs. Black/Hispanic-Black vs. Hispanic/non-Black), smoke exposure (yes vs. no as described in manuscript), and maternal education (less than high school degree vs. high school degree or more). As indicates arsenic; Ba, barium; Cd, cadmium; Cr, chromium; Cs, cesium; IBQ-R, Infant Behavior Questionnaire-Revised; Pb, lead; Sb, antimony.

As illustrated by Figure 1, multivariable WQS regression models showed a significant positive association with the Fear scale; with each decile increase in the metal mixture, scores increased by 0.20 units (95% CI: 0.09 to 0.30) (for effect estimates, see eTable 3; http://links.lww.com/EE/A131). Stratified weights revealed that girls (overall weight: 61.6%) contributed more to the mixture effect than boys (38.4%). When considering the component metals, the estimated weights for As (boys: 2.4%, girls: 12.2%), Ba (boys: 8.5%, girls: 14.0%), and Cs (boys: 3.7%, girls: 11.2%) noticeably differed by sex (see Figure 2 and eTable 3; http://links.lww.com/EE/A131). Sex-differential slopes were also identified by including a cross-product term between sex and the metal mixture, which showed a significant interaction (95% CI for cross-product term: −0.19 to −0.01). For context, infants prenatally exposed to tobacco smoke in this cohort scored 0.13 (95% CI: −0.10 to 0.37) points higher on the Fear scale compared with those unexposed and each additional year of maternal age at delivery was associated with a 0.07 (95% CI: −0.03 to 0.10) point lower Fear score. Results were in the expected direction for the global and other subdomain scales (see Figure 1 and eTable 4; http://links.lww.com/EE/A131), but stratified weights did not vary substantially by sex (see Figure 2). Weight uncertainty plots that characterize the variability in weights over repeated holdouts are provided in the Supplemental Digital Content; http://links.lww.com/EE/A131 (see Figure 4A–E). Maternal fish intake was not associated with infant scores on the Global Negative Affectivity scale or any of its subdomains (all *P* > 0.3).

## Discussion

In this study, we found prenatal exposure to a mixture of seven metals was associated with significantly increased scores on the temperamental domain of fear in infants, with girls showing particular sensitivity, and As, Ba, and Cs contributing the greatest weight to the mixture effect. Associations between the metal mixture and other domains of negative affect were in the hypothesized direction but did not reach statistical significance. The IBQ-R Fear scale captures behavioral inhibition, which is characterized by startle or distress to sudden changes in the environment and inhibited approach to novelty. This finding has important public health implications, as behavioral inhibition has been shown to be an early precursor of social anxiety and other anxiety disorders,^75,76^ as well as depression.^77,78^ While little research has examined metals exposure in relation to fear or behavioral inhibition, both metals and temperament have been linked to anxious and depressive symptomology, although human research examining the prenatal period is limited. In healthy mice, subchronic As exposure has been shown to enhance “anxiety-like” behaviors,^79^ although other research has shown prenatal As to be associated with reduced fear in male, but not female, mice.^80^ Similar research conducted in rats has shown prenatal exposure to low levels of Pb and Cd is associated with increased displays of anxious behavior.^81^ In humans, higher exposure to Cd,^82^ Pb,^83,84^ and mercury^85^ has been positively associated with depression. Little research has considered the individual or joint effects of the other metals considered here on early displays of elevated fear or its psychopathological sequela. Most notably, little research has examined Ba in relation to neurodevelopmental outcomes. Humans are primarily exposed to Ba through drinking water, household products such as depilatory creams, and foods such as nuts and seaweed.^16^ Ba sulfates are insoluble, poorly absorbed, and used as a radiologic contrast agent.^16^ Recent reports suggest Ba exposure may be increasing as a consequence of increased release from industrial processes.^15^ Ba is chemically similar to calcium (Ca) and is a bone-seeking element, which may also concentrate in teeth.^86^ The interactions with Ca++ metabolic pathways are believed to underlie its toxicity, including inhibition of potassium channels.^87,88^ It has been established that Ba accumulates in the placenta and is associated with neonatal death at high doses, but data on the mechanisms of Ba toxicity at low-dose exposure levels are limited.

Behavioral inhibition and fear are hypothesized to reflect a lower threshold of limbic excitability and higher reactivity of amygdala nuclei.^89^ The amygdala and connected subcortical and cortical brain regions play a central role in the early development of fear, including in response to novel stimuli,^90,91^ with primate research demonstrating a sex-dependent role of the amygdala in the development of behavioral reactivity to threatening situations.^92,93^ It is thus plausible that our observed findings relate to metal-induced disrupted programming of amygdala or other limbic circuits during prenatal development, although we acknowledge that research directly supporting this hypothesis is limited. One prior study showed that young mice exposed to a high-fat diet in combination with aluminum, copper, Pb, and Cd in drinking water had significantly increased “anxiety-like” behavior and impaired fear memory, with increased oxidative stress and metal accumulation recorded in the amygdala, among other brain structures.^94^ Other research suggests that gestational exposure to metals can alter the fetal and placental epigenome, including in regions related to neurodevelopment and abnormal neurobehavior.^95–100^ For example, gestational exposure to several metals has been shown to increase methylation of the placental glucocorticoid receptor (*NR3C1*), which in turn has been associated with neonatal habituation (i.e., the ability to acclimate to environmental stimuli)^101^—an early indicator of fear responding in infants.^102^ Notably, higher prenatal exposure to cortisol, a stress hormone that binds the glucocorticoid receptor, has been associated with sex-differential amygdala volumes and affective problems in girls, supporting a putative role for this receptor in the development of sex-specific fear circuitry. Unfortunately, in the PRISM cohort, maternal hair cortisol levels were measured for a small subset (n = 118) of those with information on metals and negative affectivity, precluding our ability to investigate associations with this stress hormone. However, future research that is designed to investigate cortisol as a mediating factor linking metals exposure with neurodevelopmental outcomes is of interest and would contribute to the field. Other research has shown significant sex differences in amygdala growth trajectories^103,104^ and activity in response to novel and fearful stimuli.^105–110^ In the present study, we found that girls displayed more fearful behaviors compared with boys and that the divergence in scores increased with increasing metals exposure. This is consistent with prior research that has demonstrated significantly higher levels of fear in girls compared with boys across laboratory assessment, maternal report, and paternal report instruments,^73,111^ as well as research reporting higher rates of anxiety disorders and neuroticism among women.^111,112^ We also note the possibility for an indirect effect through gestational age at birth. While the current study was not designed to formally examine mediation, prior research has linked metals exposure with preterm birth,^113^ which in turn has been associated with higher infant negative affectivity^114^ and later childhood anxiety.^115^ An interesting future direction would be to explore the role of gestational duration in mediating the associations observed here.

Strengths of this study include the prospective design and relatively large, sociodemographically diverse sample. Our statistical approach leveraged two recently developed WQS regression extensions that enabled us to (1) evaluate sex interactions and estimate stratified weights, and (2) stabilize weights across training and testing partitions and characterize uncertainty in our estimates. With regard to our outcome measure, we assessed infant negative effect using the IBQ-R, a validated and widely used research instrument that leverages the mother’s ability to observe specific infant behaviors across a range of contexts. Temperamental traits, which are relatively stable across the life course and predictive of childhood and adult psychopathologies, are a particularly useful characteristic to study in relation to prenatal exposures as they can be measured early in life, thereby minimizing the potential influence of competing risks.

There are also several notable limitations of this study. We measured maternal metal concentrations in urine, which reflects exposure from multiple sources; however, this approach is limited in that maternal levels do not directly indicate fetal exposure. Additionally, while urinary Cd and Cs are considered acceptable biomarkers of long-term exposure, urinary concentrations of the other metals reflect recent exposure only and thus may not provide a measure of average exposure across the course of pregnancy. Measuring shorter half-life metals at multiple time points would provide a more comprehensive picture of prenatal exposure; however, as with most environmental epidemiologic research, this was not feasible in the PRISM study. Further, we acknowledge that while urine is an acceptable matrix for measuring As, Ba, Cd, Cs, Cr, and Sb, whole blood is considered the gold-standard matrix for Pb. Future research that integrates exposure biomarkers across multiple matrices (e.g., Cd in urine, Pb in blood) will advance the field and is needed to substantiate our findings. Additionally, there could be exposure misclassification in our measure of As, as total urinary As reflects both toxic species as well as nontoxic arsenobetaine arising from the ingestion of seafood. This could influence our results if maternal seafood consumption relates to the development of infant temperamental traits, which was not the case in this sample and which we are not aware has been previously investigated by other studies. We were also unable to measure mercury (Hg) levels with our assay. This is unfortunate as Hg, which humans are largely exposed to through the consumption of fish, is an established developmental neurotoxicant.^116^ With regard to our modeling approach, WQS regression includes a directionality constraint, such that it tests only for mixture effects positively or negatively associated with the outcome. Given this constraint, we restricted our models to include only those metals we hypothesized to be associated with worse negative affect. However, we note that many metals are essential micronutrients that have been shown to play important roles in neurodevelopment. Unfortunately, a limitation of our approach was that we were not able to consider the combined effect of toxic and essential elements that may act in opposing directions. Finally, the sample comprised largely lower income, minority women and their infants, who are at elevated risk for metals exposure; therefore, results may not be fully generalizable to other populations or settings with lower exposure levels.

To our knowledge, this is the first study to examine associations between prenatal metal mixtures and infant negative affect. Overall, findings were consistent in direction across the global and subdomain scales considered, with the strongest associations identified with the subdomain of fear, especially among girls. This finding has important public health implications, as higher fear and behavioral inhibition during infancy show developmental continuity with a range of anxiety disorders that, in turn, carry substantial individual and societal costs.^117,118^ Thus, given the ubiquity of metal exposures and the lifelong consequences associated with heightened fear behavior and behavioral inhibition, these findings have broad implications for public health and wellbeing. Identifying and reducing major sources of metals exposure among pregnant women, particularly lower income, minority women, who are often understudied and at risk for elevated exposure, provides a potential means for improving children’s neurobehavioral development.

## Acknowledgments

We would like to acknowledge Mahmoud Awawda at the Mount Sinai Lautenberg Health Science Laboratory for performing elemental analysis and Shirisha Yelamanchili at the Mount Sinai HHEAR laboratory hub, who performed the measurements of creatinine in urine.

## Supplementary Material


