# Supplementary material for: Prenatal metal mixtures and sex-specific infant negative affectivity
Source: Environ Epidemiol. 2021 Apr 2;5(2):e147. doi: 10.1097/EE9.0000000000000147 (PMC8043734; doi:10.1097/EE9.0000000000000147)
Supplement: Supplementary file 1 [file ee9-5-e147-s001.docx]

**SUPPLEMENTAL DIGITAL CONTENT**

Prenatal metal mixtures and sex-specific infant negative affectivity

Whitney Cowell, Elena Colicino, Yuri Levin-Schwartz, Michelle Bosquet Enlow, Chitra Amarasiriwardena, Syam S. Andra, Chris Gennings, Robert O. Wright, Rosalind J. Wright

**Contents:**

Supplemental Digital Content Figure 1. Directed Acyclic Graph of assumed conditional dependencies between the exposure, outcome, and covariates.

Supplemental Digital Content Table 1. Comparison of PRISM sample characteristics among participants included versus excluded from the analytic sample.

Supplemental Digital Content Figure 2. Distribution of maternal urinary metals concentrations during pregnancy.

Supplemental Digital Content Figure 3. Comparison of metal concentrations in the PRISM (2011-2018) sample compared to NHANES data from 2015-2016.

Supplemental Digital Content Table 2. Spearman correlation coefficients (p-value) between urinary metals.

Supplemental Digital Content Table 3. Change in IBQ-R Negative Affectivity global and subscale scores per 1-log ng/mL increase in urinary metal concentrations.

Supplemental Digital Content Table 4. Change in IBQ-R Negative Affectivity global and subscale scores per 1-decile increase in the WQS Index and associated sex-specific weights for each metal.

Supplemental Digital Content Figure 4 A-E. Weight uncertainty plots of WQS mixture weights across repeated holdout validation sets for each Negative Affectivity scale.

**Figure 1**. Directed Acyclic Graph illustrating implied conditional dependencies among study variables.


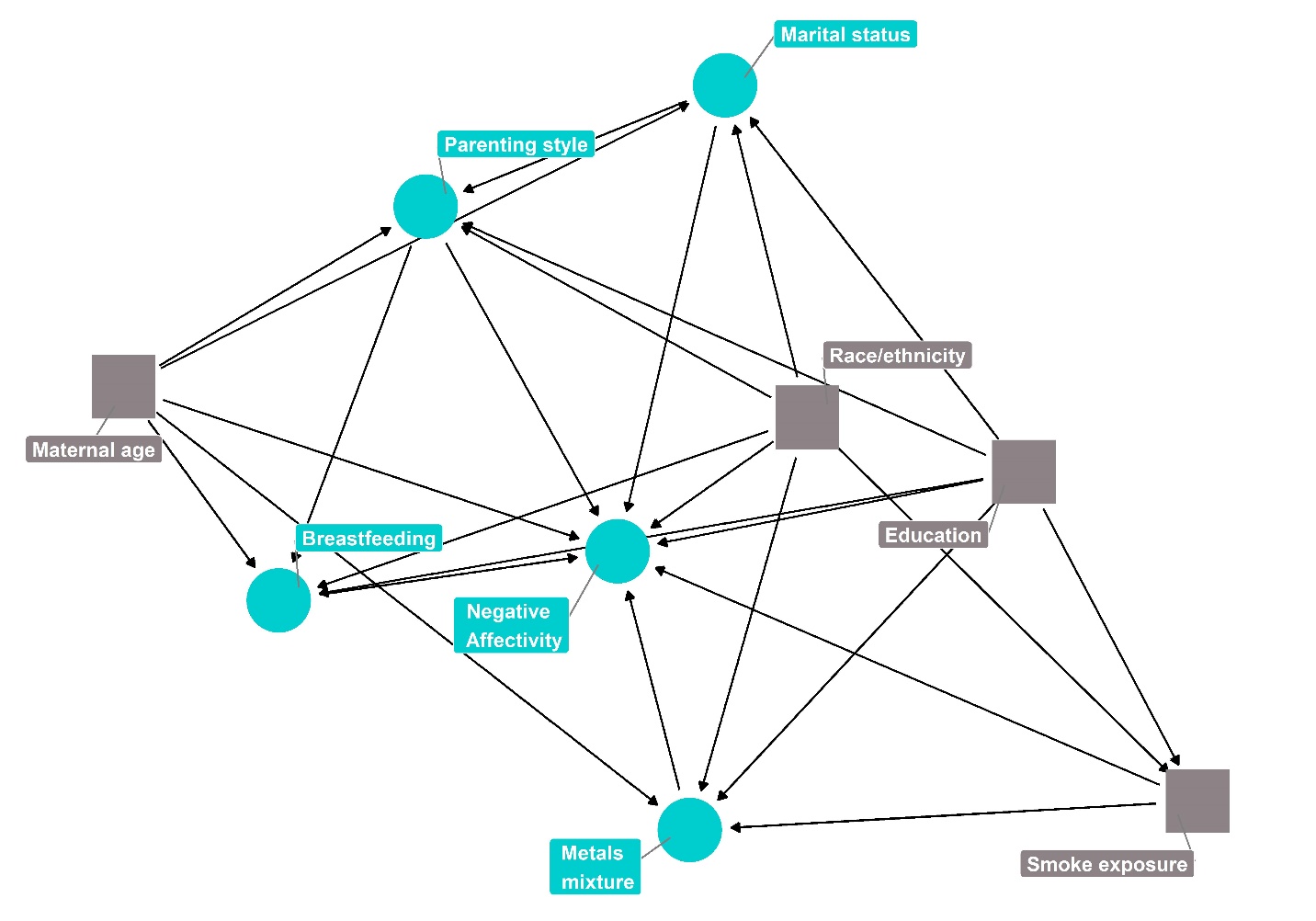


Grey squares represent variables in the minimally sufficient adjustment set (maternal education, race/ethnicity, smoke exposure, and age) that close biasing paths when controlled for. DAG created in DAGitty v3.0 (Textor 2016), and plot produced using the associated ggdag R package (Barrett 2020).

Textor, H, van der Zander B, Gilthorpe MK, Liskiewicz M, Ellison GTH. Robust causal inference using directed acyclic grapgs: the R package ‘dagitty’. *International Journal of Epidemiology* 45(6):1887-1894, 2016.

Barrett, M. ggdag: Analyze and Create Elegant Directed Acyclic Graphs. R package version 0.2.2. Published 2020.

| **Table 1.** Comparison of PRISM sample characteristics among participants included in versus excluded from the analytic sample. | | | | | |
| --- | --- | --- | --- | --- | --- |
|  | Included (N=308) | | Excluded (N=729) | | p-value^a^ |
|  | Mean±SD or  N (%) | N | Mean±SD or  N (%) | N |  |
| Maternal age (years) | 28.9±5.7 | 308 | 29.3±5.9 | 729 | 0.37 |
| Race/ethnicity |  | 308 |  | 640 | 0.0006 |
| White, non-Hispanic | 39 (12.7) |  | 116 (18.1) |  |  |
| Black, Black-Hispanic | 165 (53.6) |  | 255 (39.8) |  |  |
| Hispanic, non-Black | 95 (30.8) |  | 234 (36.6) |  |  |
| Other | 9 (2.9) |  | 35 (5.5) |  |  |
| Less than high school education | 125 (40.5) | 308 | 254 (40.1) | 634 |  |
| Smoke exposure^b^ | 121 (39.3) | 308 | 154 (23.7) | 649 | <0.0001 |
| Infant age at assessment (months) | 6.5±1.6 | 308 | 6.3±1.2 | 340 | 0.31 |
| Gestational week of urine collection | 31.1±6.0 | 308 | 31.8±5.8 | 121 | 0.27 |
| Maternal urinary creatinine (mg/dL)^c^ | 108.57 (90.45) | 308 | 88.74 (92.34) | 122 | 0.005 |
| Maternal urinary metals (ng/mL)^c^ |  |  |  |  |  |
| Antimony | 0.12 (0.09) | 308 | 0.12 (0.11) | 122 | 0.99 |
| Arsenic | 11.03 (14.67) | 308 | 12.14 (14.47) | 122 | 0.65 |
| Barium | 2.79 (3.09) | 308 | 2.66 (4.05) | 122 | 0.54 |
| Cadmium | 0.20 (0.27) | 308 | 0.21 (0.26) | 122 | 0.54 |
| Chromium | 0.62 (0.36) | 308 | 0.60 (0.27) | 122 | 0.79 |
| Cesium | 4.66 (3.68) | 308 | 4.51 (3.82) | 122 | 0.63 |
| Lead | 0.67 (0.49) | 308 | 0.67 (0.56) | 122 | 0.94 |
| IBQ-R scales |  |  |  |  |  |
| Negative Affectivity | 3.14±0.68 | 308 | 3.10±0.72 | 344 | 0.49 |
| Fear | 2.79±1.03 | 308 | 2.65±1.16 | 344 | 0.12 |
| Sadness | 3.16±0.88 | 308 | 3.23±0.99 | 344 | 0.34 |
| Distress to Limitations | 3.68±0.91 | 308 | 3.66±0.93 | 344 | 0.79 |
| Falling Reactivity | 5.07±1.03 | 308 | 5.14±0.92 | 344 | 0.38 |
| ^a^P-values are from t-tests (continuous variables) or chi-square tests of homogeneity (categorical variables) unless otherwise noted.  ^b^Defined as maternal cigarette, cigar, or pipe smoking during pregnancy or exposure to environmental tobacco smoke for 1-hour or more per week during pregnancy.  ^c^Summary statistics are geometric mean (interquartile range), and p-values are from Wilcoxon Rank Sum tests. | | | | | |

**Figure 2.** Distribution of maternal urinary metal concentrations during pregnancy.


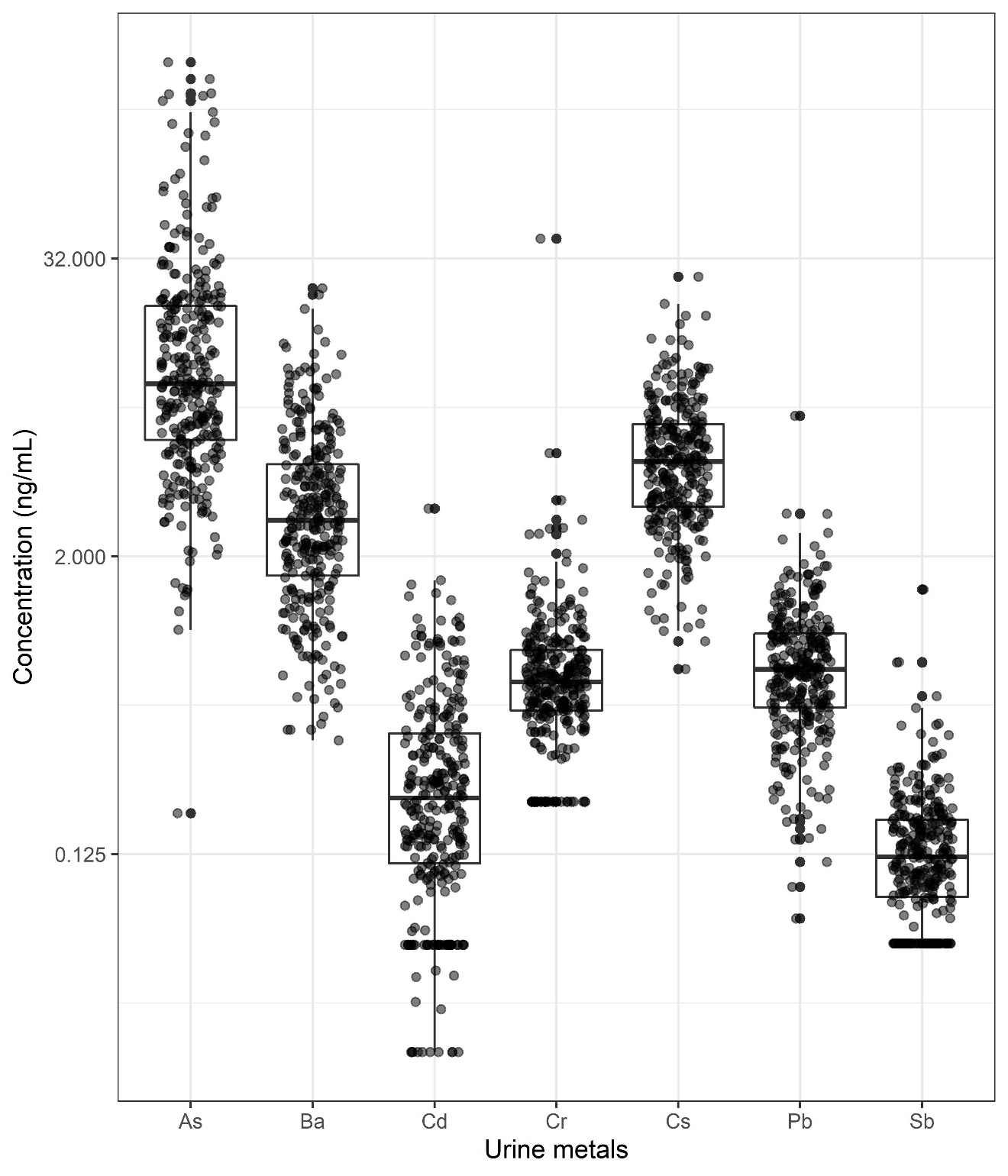


**Figure 3**. Urinary metal concentrations in the Programming of Intergenerational Stress Mechanisms (PRISM) sample compared with National Health and Nutrition Examination Survey (NHANES) data.


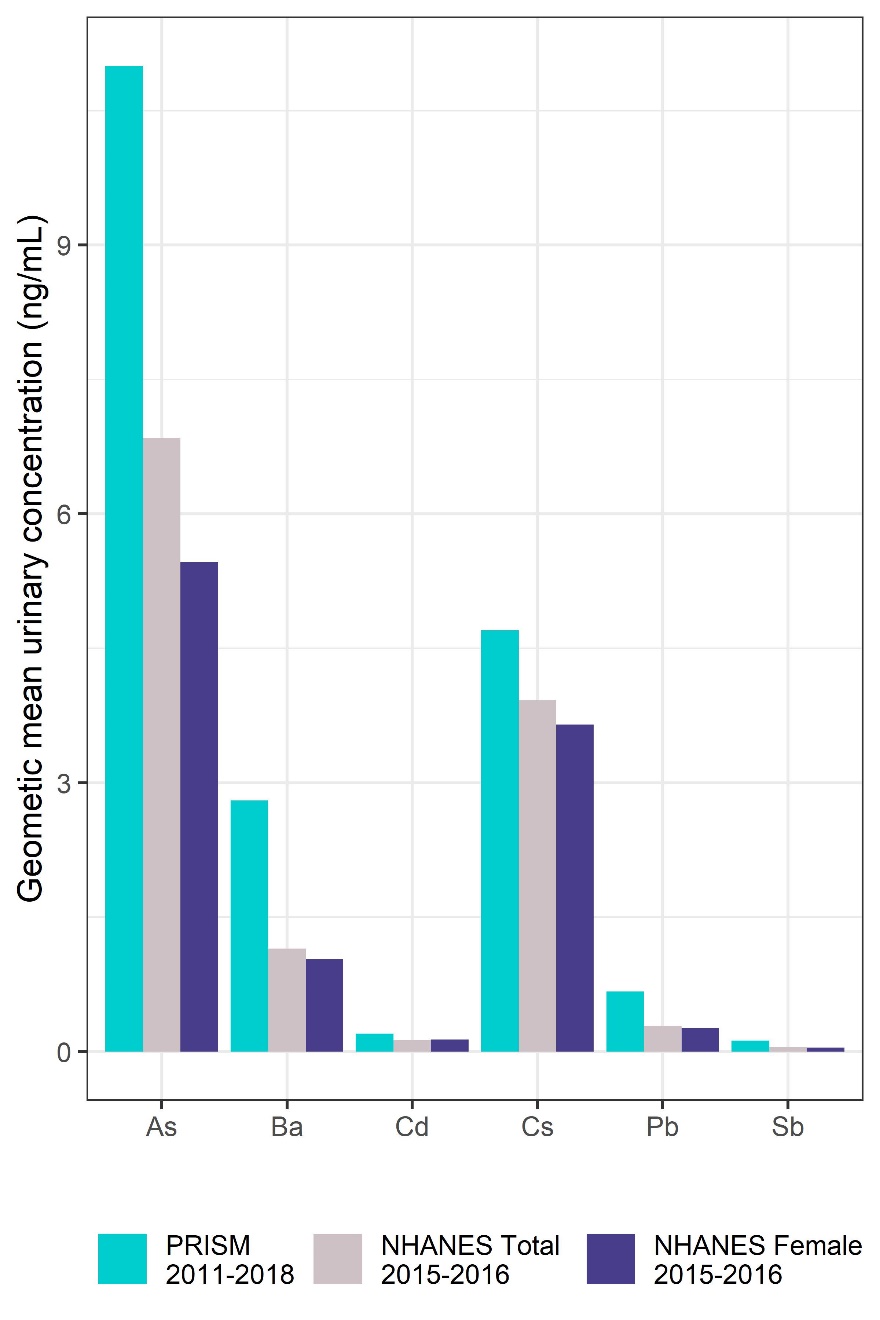


Legend: Chromium is excluded, as urinary concentrations are not measured by NHANES. Abbreviations: As, arsenic; Ba, barium; Cd, cadmium; Cr, chromium; Cs, cesium; Pb, lead; Sb, antimony.

| **Table 2.** Spearman correlation coefficients (p-value) between urinary metals | | | | | | | | |
| --- | --- | --- | --- | --- | --- | --- | --- | --- |
|  | As | Ba | Cd | Cr | Cs | Pb | Ni | Sb |
| As | 1.00 | 0.19 (0.0007) | 0.29 (<0.0001) | 0.16 (0.0041) | 0.41 (<0.0001) | 0.26 (<0.0001) | 0.32 (<0.0001) | 0.21 (0.0002) |
| Ba |  | 1.00 | 0.26 (<0.0001) | 0.19 (0.0008) | 0.28 (<0.0001) | 0.37 (<0.0001) | 0.32 (<0.0001) | 0.23 (<0.0001) |
| Cd |  |  | 1.00 | 0.38 (<0.0001) | 0.64 (<0.0001) | 0.61 (<0.0001) | 0.52 (<0.0001) | 0.48 (<0.0001) |
| Cr |  |  |  | 1.00 | 0.34 (<0.0001) | 0.51 (<0.0001) | 0.44 (<0.0001) | 0.43 (<0.0001) |
| Cs |  |  |  |  | 1.00 | 0.53 (<0.0001) | 0.54 (<0.0001) | 0.43 (<0.0001) |
| Pb |  |  |  |  |  | 1.00 | 0.44 (<0.0001) | 0.54 (<0.0001) |
| Ni |  |  |  |  |  |  | 1.00 | 0.36 (<0.0001) |
| Sb |  |  |  |  |  |  |  | 1.00 |

Abbreviations: As, arsenic; Ba, barium; Cd, cadmium; Cr, chromium; Cs, cesium; Pb, lead; Sb, antimony.

| **Table 3.** Change in IBQ-R negative affect global and subscale scores per 1-log ng/mL increase in urinary metal concentrations. Reported values are β (95% CI). | | | | | |
| --- | --- | --- | --- | --- | --- |
|  | Negative Affectivity Global | Fear | Sadness | Distress to Limitations | Falling Reactivity |
| As | 0.016  (-0.063, 0.095) | 0.044  (-0.074, 0.163) | 0.021  (-0.084, 0.126) | 0.022  (-0.083, 0.128) | 0.024  (-0.098, 0.146) |
| Ba | 0.100  (0.008, 0.192) | 0.167  (0.029, 0.306) | 0.123  (0.000, 0.246) | 0.015  (-0.110, 0.139) | -0.095  (-0.239, 0.048) |
| Cd | 0.104  (0.002, 0.207) | 0.114  (-0.041, 0.268) | 0.063  (-0.074, 0.200) | 0.122  (-0.015, 0.259) | -0.119  (-0.278, 0.040) |
| Cr | 0.000  (-0.135, 0.135) | 0.018  (-0.186, 0.222) | 0.024  (-0.156, 0.204) | -0.049  (-0.231, 0.132) | -0.008  (-0.218, 0.202) |
| Cs | 0.112  (-0.047, 0.271) | 0.201  (-0.039, 0.441) | 0.153  (-0.059, 0.365) | 0.058  (-0.156, 0.272) | -0.036  (-0.283, 0.212) |
| Pb | 0.015  (-0.132, 0.151) | 0.187  (-0.032, 0.406) | -0.010  (-0.204, 0.185) | -0.101  (-0.296, 0.095) | -0.135  (-0.361, 0.091) |
| Sb | 0.002  (-0.149, 0.154) | 0.151  (-0.076, 0.378) | -0.087  (-0.288, 0.114) | 0.004  (-0.199, 0.207) | 0.059  (-0.176, 0.293) |
| Abbreviations: As, arsenic; Ba, barium; Cd, cadmium; Cr, chromium; Cs, cesium; Pb, lead; Sb, antimony.  Adjusted for: urinary creatinine, gestational week at urine collection, infant age at assessment, maternal age, education, race/ethnicity, and tobacco smoke exposure during pregnancy. | | | | | |

| **Table 4.** Change in IBQ-R negative affect global and subscale scores per 1-decile increase in the WQS Index and associated sex-specific weights for each metal. | | | | | | | | | | |
| --- | --- | --- | --- | --- | --- | --- | --- | --- | --- | --- |
|  | Negative Affectivity Global | | Fear | | Sadness | | Distress to Limitations | | Falling Reactivity | |
| β (95% CI) | 0.07 (-0.01, 0.14) | | 0.20 (0.09, 0.30) | | 0.06 (-0.04, 0.16) | | 0.02 (-0.08, 0.12) | | -0.02 (-0.13, 0.09) | |
| Total Weights (%) | Boy | Girl | Boy | Girl | Boy | Girl | Boy | Girl | Boy | Girl |
| As | 6.7 | 6.1 | 2.4 | 12.2 | 11.0 | 3.7 | 10.8 | 5.7 | 7.0 | 5.2 |
| Ba | 9.9 | 14.7 | 8.5 | 14.0 | 10.8 | 14.9 | 5.5 | 7.2 | 5.7 | 13.3 |
| Cd | 12.4 | 10.8 | 5.3 | 7.2 | 8.8 | 5.9 | 15.7 | 12.1 | 15.0 | 11.9 |
| Cr | 3.5 | 2.9 | 4.3 | 4.6 | 1.2 | 7.2 | 2.4 | 2.2 | 5.3 | 5.1 |
| Cs | 2.5 | 14.7 | 3.7 | 11.2 | 4.8 | 15.2 | 1.9 | 15.2 | 5.0 | 8.1 |
| Pb | 4.8 | 2.4 | 6.8 | 6.8 | 7.2 | 2.5 | 4.2 | 4.3 | 2.9 | 1.8 |
| Sb | 6.6 | 2.1 | 7.4 | 5.7 | 3.5 | 3.4 | 8.4 | 4.1 | 12.4 | 1.3 |
| Total | 46.4 | 53.8 | 38.4 | 61.6 | 47.2 | 52.8 | 49.0 | 51.0 | 53.3 | 46.7 |
| Abbreviations: As, arsenic; Ba, barium; Cd, cadmium; Cr, chromium; Cs, cesium; Pb, lead; Sb, antimony.  Adjusted for: urinary creatinine, gestational week at urine collection, infant age at assessment, maternal age, education, race/ethnicity, and tobacco smoke exposure during pregnancy.  Estimates are interpreted as the change in IBQ-R score for a 1-decile increase in the metal mixture, and weights reflect the component contribution to the overall mixture effect. The presented parameter estimates and weights reflect the mean values from the 100 repeated models. | | | | | | | | | | |

**Figure 4 A-E:** Weight uncertainty plots depicting weighted quantile sum mixture weights (y-axis) for each of 100 repeated holdout validation sets.


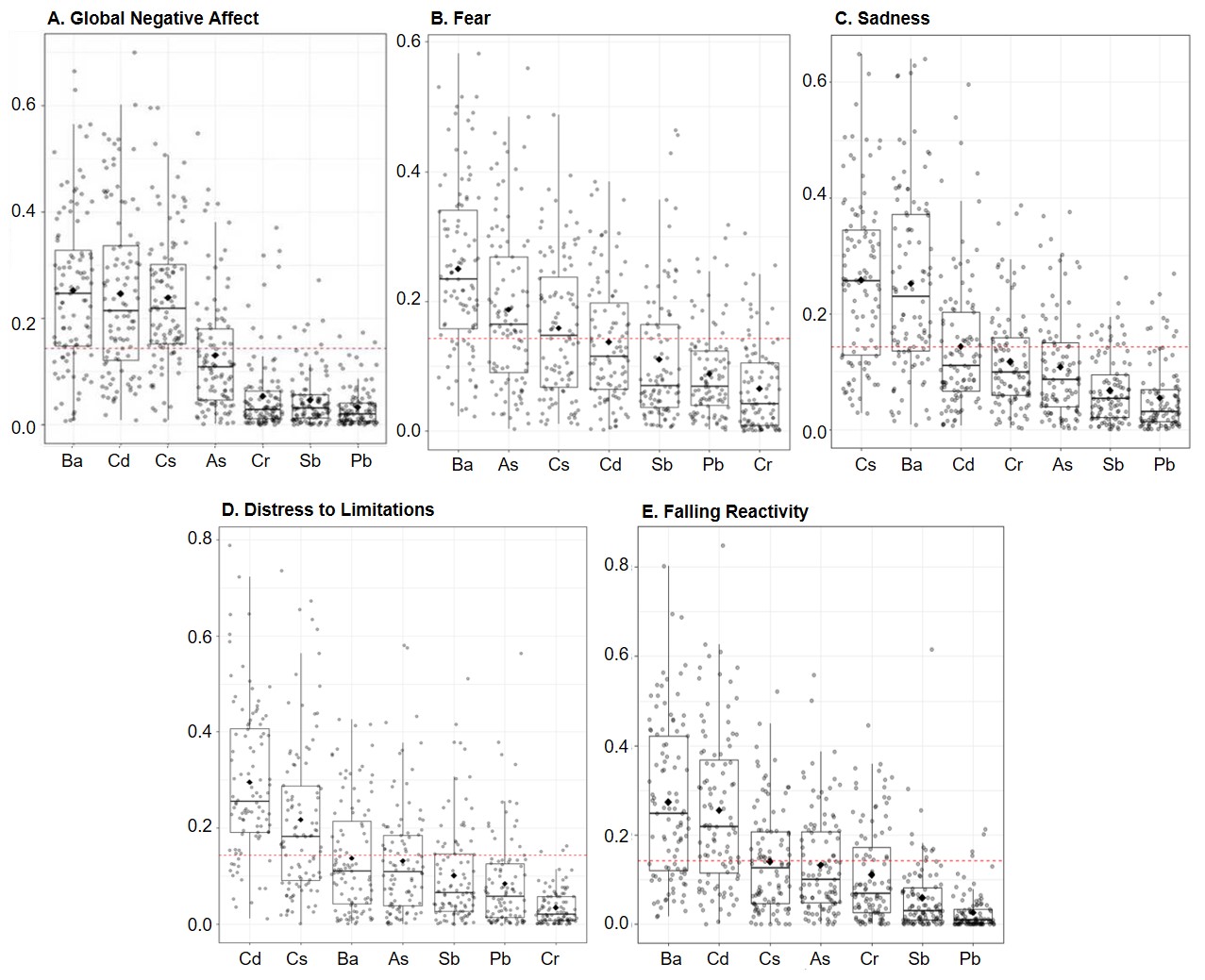


Abbreviations: As= arsenic, Ba=barium, Cd=cadmium, Cs = cesium, Cr = chromium, Pb=lead, Sb = antimony, WQS=weighted quantile sum. Notes: The red lines indicate the concern threshold of 14.3% in 100 repeated holdouts. Data points indicate weights for each of the 100 holdouts. Box plots show 25^th^, 50^th^, and 75^th^ percentiles, and whiskers show 10^th^ and 90^th^ percentiles of weights for the 100 holdouts. Closed diamonds show mean weights for the 100 holdouts. Adjusted for: urinary creatinine, gestational week at urine collection, infant age at assessment, maternal age, education, race/ethnicity, and tobacco smoke exposure during pregnancy.
